# Comparison of qualitative and semiquantitative strain elastography in breast lesions for diagnostic accuracy

**DOI:** 10.1186/s40644-016-0070-8

**Published:** 2016-05-26

**Authors:** Timothy Musila Mutala, Purity Ndaiga, Angeline Aywak

**Affiliations:** Department of Diagnostic Imaging and Radiation Medicine, College of Health Sciences, University of Nairobi, Kenyatta National Hospital, P. O. Box 19676-00202, Nairobi, Kenya

**Keywords:** Semiquantitative elastography, Breast solid masses, Benign, Malignant, Strain ratio, Strain score

## Abstract

**Background:**

Strain elastography can be purely qualitative or semiquantitative using both strain score and strain ratio. The aim of this study was to establish the accuracy of semiquantitative elastography using both strain score and strain ratio in differentiating benign from malignant breast masses. The diagnostic performance of the two methods was analysed for any statistically significant difference.

**Methods:**

A prospective study was carried out from May to December 2014 in the University of Nairobi, Department of Diagnostic Imaging and Radiation Medicine. One hundred and eighteen patients referred for breast ultrasound following clinical detection of masses certified the inclusion criteria. All solid masses identified on grey scale imaging were subjected to strain elastography. Elastographic findings were represented in both strain score and strain ratio. Comparison of diagnostic performance with histological findings as the gold standard for all detected solid masses was done. Fisher’s exact test and receiver operating characteristics curves were applied for statistical analysis to look for any significant differences between the diagnostic performance of strain score and strain ratio.

**Results:**

Out of the 118, three patients did not attend for all the examinations and three biopsy results were misplaced therefore analysis was done for 112 subjects. The sensitivity, specificity, positive predictive value and negative predictive value of elasticity strain (Ueno) score were 0.86, 0.96, 0.89 and 0.96 respectively. For the strain ratio the values were 0.93, 0.96, 0.90 and 0.96 respectively. Fisher’s exact test P values comparing the sensitivity and specificity were 0.69 and 1.00 respectively not considered significant at p 0.05 levels. The areas under the curve (AUCs) from the receiver operating characteristic (ROC) curves were 0.972 and 0.976 for strain score and ratio respectively with a strong Pearson’s correlation coefficient, r 0.79 indicating a high diagnostic accuracy for both methods but no statistically significant difference in performance.

**Conclusion:**

Semiquantitative ultrasound elastography has good diagnostic accuracy in differentiating benign and malignant breast solid lesions and there is no statistically significant difference between strain score and strain ratio in sensitivity, specificity and accuracy.

**Electronic supplementary material:**

The online version of this article (doi:10.1186/s40644-016-0070-8) contains supplementary material, which is available to authorized users.

## Background

Breast cancer is the commonest cancer in women both in developed and developing world. Statistics in Kenya indicate that breast cancer contributes to 23.3 % of cancer burden [1]. Mammography screening has gained recognition for its significant mortality reduction especially in the developed world even though screening programs are not in place in many sub-Saharan African countries including Kenya. Ultrasound has a complementary role to mammography in breast cancer diagnosis by preventing unnecessary biopsies and short term follow up of mammographic benign lesions, guiding interventions and giving feedback that improves clinical and mammographic skills with intent to better early detection [2]. At the same time in younger patients as well as pregnant women ultrasound is the preferred method of choice in lesion detection and characterisation [3]. Furthermore ultrasound has been recommended as a screening tool for high risk women population like known BRCA1 or BRCA2 mutations or those with close relatives affected by breast cancer before menopause especially in a setting where MRI is not feasible [4]. Grey scale sonography has assigned characteristics that grade the probability of a breast solid mass being either benign or malignant according to the breast imaging reporting and data systems (BI-RADS). These include shape, surface and internal characteristics of the lesion which have been described to yield a sensitivity and negative predictive value of 98.4 and 99.5 % respectively in the best of hands [5].

Ultrasound elastography is an extension of clinical palpation based on the fact that malignant lesions are stiffer than their benign counterparts. Using elastography, tissue stiffness (or hardness) can be measured and converted into an image. It has been used to increase diagnostic accuracy by reducing the number of false positives on B mode ultrasound therefore obviating unnecessary biopsies [6]. The physics behind this principle relies on tissue stiffness quantified by Young’s modulus (E or elasticity). Young’s modulus (elasticity) = Stress/Strain or E = s/e. If the amount of force (stress) initially applied to tissue is known, elasticity can be determined. Elasticity (E) is measured in pressure units, pascals, or kilopascals (kPa). Most cancers feel stiffer on palpation because they have a lower strain value and a higher Young’s modulus. Ultrasound elastography utilizes either strain or shear- wave elastography. Strain elastography is also known as static or compression elastography. With this technique, gentle repetitive compression is applied to tissue with an ultrasound probe or natural motion (e.g. heartbeat or respiration). Strain is greater in soft tissue compared to hard tissue because soft tissue will easily deform when subjected to external pressure. Strain elastography provides qualitative information through the elasticity (Ueno or Tsukuba) scores. Due to the challenge of inter-observer variability semiquantitative assessment was introduced through the application of strain ratios that are calculated by comparing average strain in a region of interest (ROI) within the lesion with that of surrounding fat tissue [7]. However strain elastography cannot provide quantitative information.

The other method is shear-wave elastography also known as transient elastography. In this technique automatic pulses generated by the ultrasound probe induce transversely oriented shear-waves within tissue. The speed of propagation of the shear-waves can be captured by the ultrasound system. This speed is directly proportional to stiffness and Young’s modulus using the formula E ≈ 3 ρ*v*^2^, where ρ = density of tissue (this is a constant in tissue at 1000 kg/m3) and *v* = shear wave propagation velocity. Shear-wave elastography provides quantitative information because elasticity of the tissue can be measured in kPa. Shear-waves travel faster in hard tissue and therefore, hard tissues will have greater kPa values compared to soft tissue.

Future potential applications of elastography include its use in characterization of small incidental masses seen on screening breast ultrasound, identification of malignant axillary lymph nodes, identification of subtle masses following MRI and specification of the more suspicious portion of a lesion to help guide ultrasound biopsy. Studies have also shown that USE has a promising role in assessing neoadjuvant chemotherapy response in breast cancer [8, 9].

It is against this background that we set out to study strain elastography and in particular compare the diagnostic accuracy of the qualitative (strain score) and semiquantitative (strain ratio) methods in a bid to reduce the number of unnecessary biopsies currently done.

## Methods

This prospective study was conducted between May and December 2014 in the University of Nairobi’s department of Diagnostic Imaging and Radiation Medicine which is located within the premises of Kenyatta National Hospital, Nairobi, Kenya. A total of 118 consecutive patients with solid breast lesions were invited to participate in the study, with 115 (97.4 %), 4 male and 111 female consenting. Both strain elasticity score and strain ratio were recorded during elastographic examination. These patients were part of a population that had been referred to the department for routine diagnostic purposes and not solely for the study. Approval by Kenyatta National Hospital and University of Nairobi ethical review committee before commencement of the study was given and the participating patients’ consent acquired before recruitment. Patients with multiple lesions had all of them studied but entry was made for the one that had the highest BI RADS status. This meant that one lesion per patient was recorded in the data spreadsheet.

The researchers had prior training in breast elastography as part of a bigger number of radiologists and sonographers who had been inducted in 2013 before the start of this study. The team was led by AA who holds ultrasound fellowship from Thomas Jefferson University in the United States of America and has wide experience in the field. To mitigate operator dependence which is a well-known pitfall in sonographic imaging the same team carried out all the examinations for this study. A high end logic E-9 GE ultrasound machine with elastography module capable of performing both strain score and ratio was consistently used for all the patients. The elastograms were displayed side by side with the conventional grey scale images. Standard care protocol in our department that involves bilateral breast examination for all patients using high frequency transducer (7–10 MHz) was applied to the subjects of this study.

**Fig. 1:**
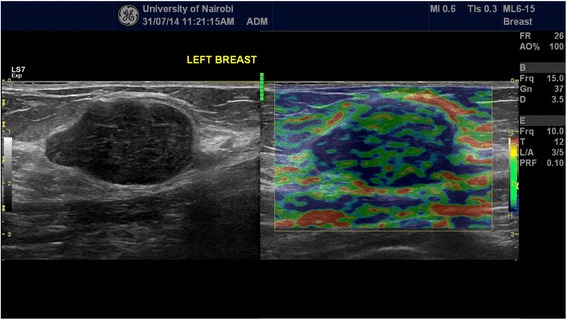
Side by side grey scale and score 2 (benign) elastogram images acquired during our study of a lesion whose histology demonstrated a fibroadenoma (benign)

**Fig. 2:**
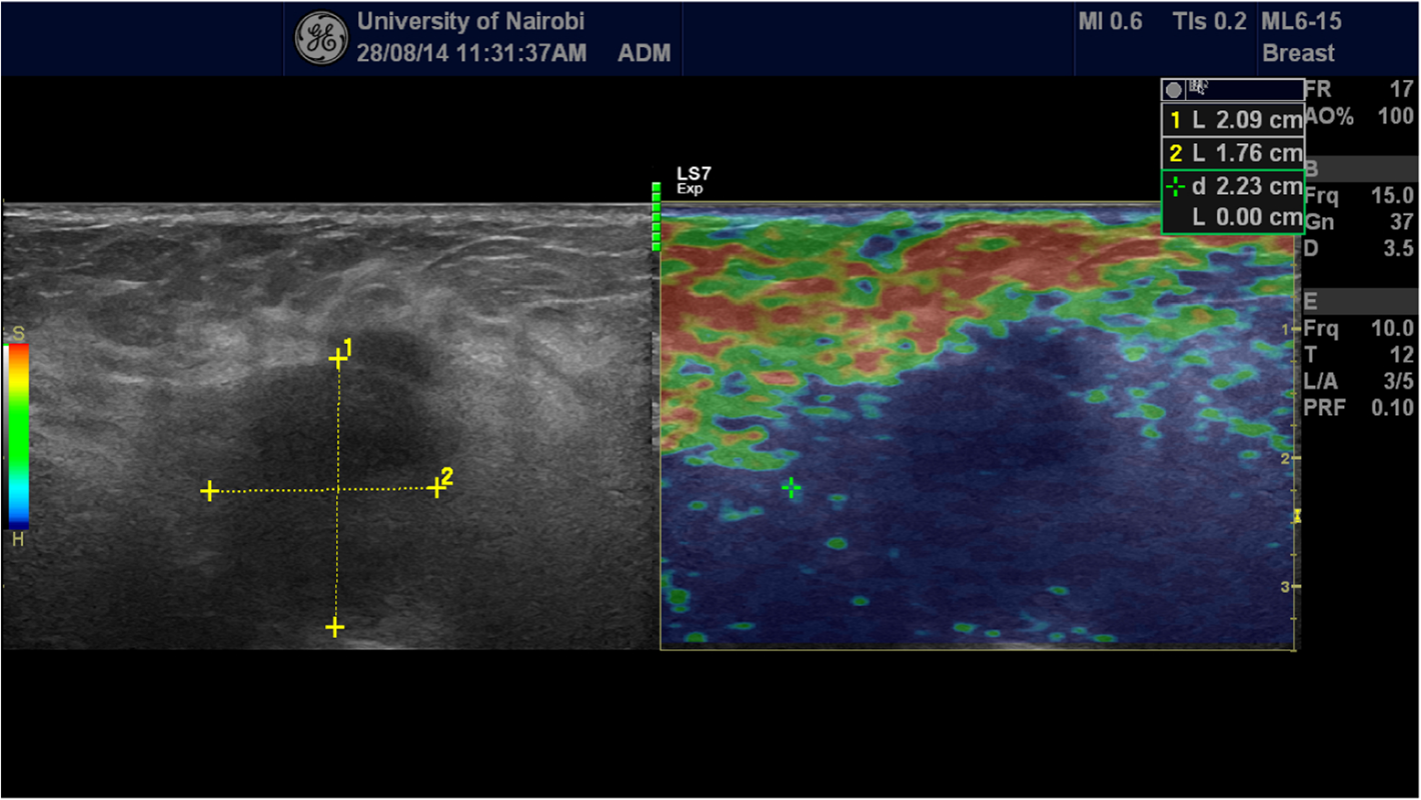
Side by side grey scale and score 5 (malignant) elastogram images of a lesion that histologically turned to be invasive ductal carcinoma (malignant)

**Fig. 3:**
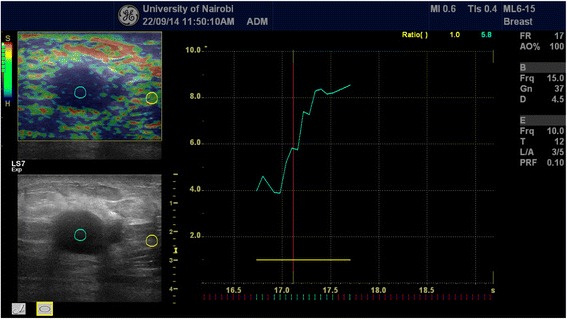
A histologically proven invasive ductal adenocarcinoma demonstrated on combined grey scale, strain score and strain ratio elastography. The strain ratio in this case was 5.8 way above the cut-off point of 4.2

Each of the solid mass lesions had an elasticity (Ueno or Tsukuba) score assigned to it and concomitant strain ratio obtained. The elastographic description of a benign lesion was given in classes 1 and 2 while malignant lesion was described by classes 4 and 5 with class 3 assigned for probably benign lesions according to the standard already published Ueno (Tsukuba) score (Figs. 1 and 2). Strain ratio was then calculated for all lesions by selecting a region of interest (ROI) on the mass and a corresponding ROI of the adjacent adipose tissue (Fig. 3). Using machine inherent software, the SR value was displayed on a static image. Being a prospective study, at the time of examination only those lesions that did not have histological diagnoses were included.

Histopathological correlation was done for 112 breast lesions. Biopsy methods included both open and percutaneous methods. Two biopsy results could not be traced while one case was deemed as inconclusive and a repeat biopsy requested later not to be captured during the study. A receiver operating characteristics (ROC) curve for each of the two methods was plotted. Sensitivity, specificity, positive and negative predictive values were derived for the strain ratio and the elasticity score separately.

Raw data was captured using a questionnaire and entries made into MS Excel© software. Data was analysed using STATA© version 11. The ROC was plotted using STATSTODO© software. Simple descriptive statistics such as means, proportions and frequency distributions with 95 % CI were used for the study sample. Fisher’s exact test and receiver operating characteristics curve areas under the curve (AUC) were applied for statistical analysis to look for any significant differences between the diagnostic performance of strain score and strain ratio.

## Results

A total of 118 patients were invited to participate in the study, with 115 (97.4 %) consenting. 112 breast lesions were confirmed by histopathology. Two biopsy results could not be traced while one case was deemed as in-conclusive and a repeat biopsy requested. The age range was 15 to 79 years with a median 28 years. The age of the patients who were diagnosed with cancer was spread between 28 and 79 years with a median age of 48 years. The median length of the masses was 2.2 cm (IQR 1.8, 3.2 cm) while the width was 1.4 cm (IQR 1.0, 2 cm). The longest mass was 7 cm, while the widest was 6 cm. The smallest mass was 1 × 0.2 cm while the largest was 7 × 4.5 cm. While elastography classified 81 lesions as benign, histologically 84 were benign. On elastography three masses which were classified as malignant on both strain score and ratio were found to be benign. The benign lesions included fibroadenomas, lipomas, papillomas, granulomatous mastitis, gynaecomastia and other non-specified benign lesions (Table 1). The remaining 28 lesions were invasive carcinomas displayed in the same table. The majority of the lesions (61.6 %) had strain score of 2 as demonstrated in Table 2. The median strain ratio of benign and malignant lesions was 1.8 and 7.2 respectively. The sensitivity, specificity, positive predictive value and negative predictive value for malignancy using the strain score were 0.86, 0.96, 0.89 and 0.96 respectively. For the strain ratio the sensitivity, specificity, positive predictive value and negative predictive value were 0.93, 0.96, 0.90 and 0.96 respectively (Table 3).

**Table 1:** Histological diagnosis of lesions

Diagnosis	Freq.	Percent
Fibroadenoma	74	66.0
Invasive ductal carcinoma	28	25.0
Benign breast lesion	2	1.7
Ductal Papilloma	2	1.7
Gynaecomastia	2	1.7
Lipoma	2	1.7
Granulomatous mastitis	1	0.9
Mastitis	1	0.9

**Table 2:** Elasticity (strain) score of the breast masses

Strain Score:	Freq.	Percent
1	2	1.8
2	69	61.6
3	14	9.0
4	11	11.6
5	16	16.0
Total	112	100.0

**Table 3:** Cross-tabulation of strain score, strain ratio and BI-RADS score against histological diagnosis. BI-RADS 1 and 2 were considered negative while 3–5 were designated positive status

		Histology positive	Histology negative
Strain score	Positive	24	3
	Negative	4	81
Strain ratio	Positive	26	3
	Negative	2	81
BI-RADS classification	Positive	28	11
	Negative	0	73

**Fig. 4:**
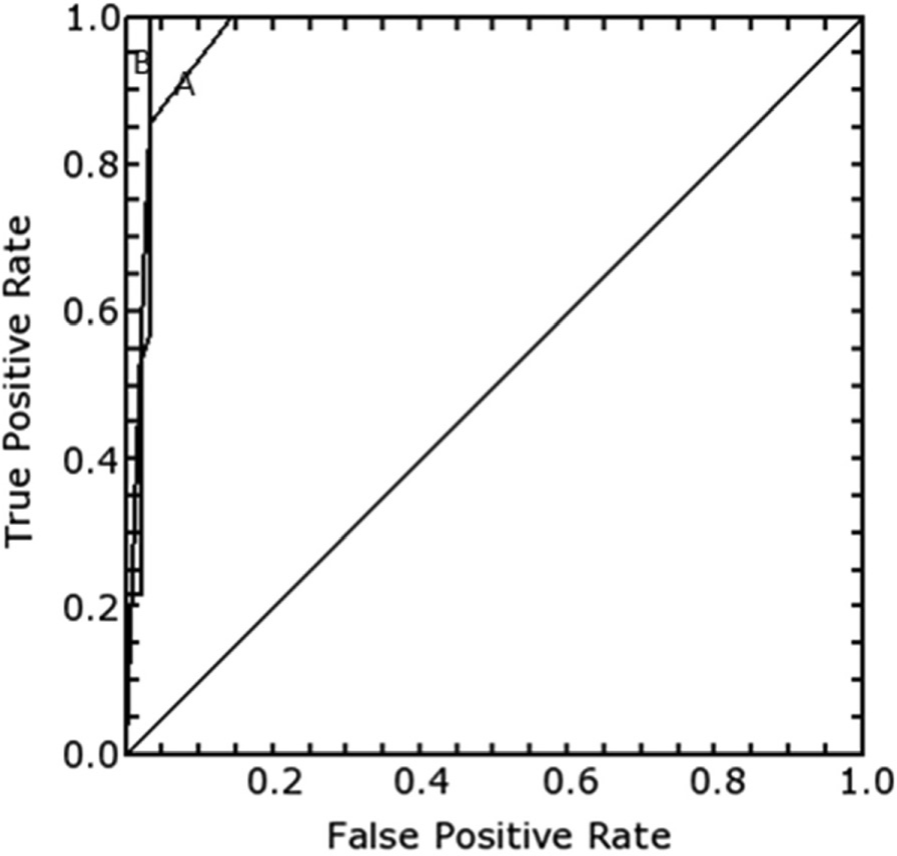
The receiver operating characteristics curves for strain score (A) and strain ratio (B)

Receiver operating characteristic (ROC) curves for both techniques were plotted (Fig. 4). The areas under the curve (AUCs) from the ROC curves were 0.972 and 0.976 for strain score and ratio respectively with a strong Pearson’s correlation coefficient, r 0.79. The difference between the two AUCs is 0.004 indicating no statistical variability on analysis (NaN value). This indicates a high diagnostic accuracy for both methods but no statistically significant difference on performance.

From the strain score ROC curve a value of three or greater was considered positive with a sensitivity of 0.86 and specificity of 0.96. For the strain ratio ROC curve a cutoff point at 4.2 gave sensitivity of 0.93 and specificity of 0.96.

Cross-tabulation of the strain score against strain ratio for positive malignant lesions on histological diagnosis was done from which comparison was deduced (Table 3). Fisher’s exact test P values comparing the sensitivity and specificity were 0.69 and 1.00 respectively not considered significant at 0.05 levels. Our study focus was not comparing diagnostic performance of grey scale BI-RADS classification against elastography, nevertheless we included a row in the same table which also did not show overall statistical significance of difference. However on case to case analysis elastography made some impact in correctly predicting the histological diagnosis in BI-RADS class 3 lesions. This explains the decline of false positive cases from 11 to 3 on grey scale and elastographic imaging respectively.

## Discussion

Combined use of grey scale ultrasonography and elastography has been documented to have higher diagnostic accuracy [10]. Strain elastography mainly provides qualitative information, although strain ratios may be calculated by comparing a lesion to the surrounding normal tissue giving semiquantitave analysis. Strain ratios have been correlated with the benignity or malignancy characteristics of lesions where lower ratios are seen with benign lesions in comparison to malignant lesions [11, 12]. Benign lesions can have reduced visibility on an elastogram while their malignant counterparts are more clearly visible due to their higher stiffness than surrounding normal tissue on an elastogram [13]. Fleury et al [14] investigated whether USE could differentiate benign from malignant breast lesions with histological correlation. The positive predictive value, specificity, and diagnostic accuracy of the scores were 76.5, 95.9, and 94.7 %, respectively. They concluded that classification by elastography can be used as an important tool combined with B mode evaluation for differentiating benign and malignant lesions of the breast. In a hospital based preliminary study done in China, Parajuly et al found that ultrasound elastography was superior in detecting breast cancer, since the accuracy (95.8 %), sensitivity (98.6 %), specificity (96.0 %), and positive predictive values (94.5 %) were higher than those of B mode sonography (90.6, 91.4, 90.0 and 86.5 % respectively) [15]. Evans et al [16] carried out a study assessing the performance of shear wave elastography combined with BI-RADS classification of gray scale images to differentiate benign and malignant breast lesions. Combination of BI-RADS gray scale and shear wave elastography yielded superior sensitivity to BI-RADS alone. All the same our study concentrated on strain elastography. A few studies have been done in Africa to assess accuracy of breast USE in differentiating benign from malignant breast masses. In Egypt Aly et al in 2009 carried out a prospective study to evaluate the accuracy of USE in distinguishing benign and malignant solid breast lesions with pathologic results as the reference standard. They reported 87.2 % sensitivity, 90.6 % specificity and 90 % accuracy and concluded that USE can facilitate improved classification of benign and malignant breast masses [17]. Our study produced higher sensitivity and specificity that could probably be attributed to advancement of ultrasonic technology between the two study periods in consideration. Multiple studies have shown that ultrasound elastography may provide additional diagnostic information to further characterize breast lesions and has the potential to improve the specificity of low suspicion lesions evaluated with conventional ultrasound. Elastography features including size ratios, shape, homogeneity and quantitative analysis may be complementary to conventional ultrasound in the comprehensive analysis of breast lesions. Clinical utility of elastography includes further evaluation of equivocal lesions on grey scale. Some definite lesions in BIRADS 4c, 5 or 2 on grey scale may not need further evaluation with elastography. Upgrading or downgrading of the lesion on elastography has been documented. Qualitative shear-wave elastography and color assessment of lesion stiffness, oval shape and a maximum elasticity value of less than 80 kPa could reduce unnecessary biopsy of low-suspicion BI-RADS 4A masses without a significant loss in sensitivity [18].

Overall diagnostic performance of breast USE from our study is excellent though no statistical difference between strain score and ratio values could be established at *p* < 0.05. This is an area that may need further interrogation in future studies. From our findings we concur with the assertion that ultrasound elastography in addition to grey scale imaging increases the confidence of categorising breast lesions within the BI-RADS lexicon especially in category 3 and can positively contribute in reducing unnecessary biopsies.

Operator dependence is a recognised pitfall of ultrasound elastography especially when using the strain method. To mitigate such possible confounding we ensured that all the elastograms were conducted by the same team that had prior training and were working together for the purpose of this study. Each member of the team did the examinations independently and where there were variations, a consensus was reached following consultations and rescanning. There is a learning curve for performing breast ultrasound elastography and performance of at least 30 examinations under supervision before gaining acceptable competence has previously been recommended [19]. Depth and size of lesion may affect the diagnostic accuracy of elastography. Some authorities state that lesions more than 3 cm in diameter may not be adequately evaluated [20]. In our experience from this study even the masses which were on the larger side of the scale did not affect the diagnostic performance of either method. Elastography correctly indicated benignity and malignancy respectively in a 7 × 4.5 cm fibroadenoma and a 5 cm ductal cancer. In fact the false positives encountered were two post-mastectomy (one partial and the other total) and one chronic granulomatous mastitis breast lesions. This can be explained by the fact that both scar and granulomatous tissues can lead to increased stiffness.

A study by Parajuly et al in China concluded that strain ratio has better diagnostic performance than elasticity score for breast elastography [21]. Findings from our study show that there is no significant difference in sensitivity, specificity and accuracy between the two methods for breast lesions. One of the greatest challenges in performing strain ratio sonoelastography will be determining the universal cut off for benignity or malignancy potential of a lesion. Just like in our study, previously published studies have varied cut offs determined through ROC curves. To that effect clustering of strain ratio values to fit into BI-RADS categories might be a useful area for future research and consensus. Probably this will mirror the already established strain score categorisation. We also encourage other sonologists and sonographers to conduct more research on this subject such that a robust database can be created for inclusion in meta- analytic studies.

## Conclusion

Semiquantitative ultrasound elastography has good diagnostic accuracy in differentiating benign and malignant breast solid lesions and there is no statistically significant difference between strain score and strain ratio in sensitivity, specificity and accuracy.

## Additional file

Additional file 1: Raw data. (XLSX 35 kb)
